# Targeting Metabolism and Autophagy in the Context of Haematologic Malignancies

**DOI:** 10.1155/2012/595976

**Published:** 2012-07-08

**Authors:** Versha Banerji, Spencer B. Gibson

**Affiliations:** ^1^Manitoba Institute of Cell Biology, 675 McDermot Avenue, Winnipeg, MB, Canada R3E 0V9; ^2^Department of Internal Medicine, Faculty of Medicine, University of Manitoba, 770 Bannatyne Avenue, Winnipeg, MB, Canada R3T 2N2; ^3^CancerCare Manitoba, Section of Haematology/Oncology, 675 McDermot Avenue, Winnipeg, MB, Canada R3E 0V9; ^4^Biochemistry and Medical Genetics, Faculty of Medicine, University of Manitoba, 745 Bannatyne Avenue, Winnipeg, MB, Canada R3E 0J9

## Abstract

Autophagy is a cellular process that maintains the homeostasis of the normal cell. It not only allows for cell survival in times of metabolic stress with nutrient recycling but also is able to lead to cell death when required. During malignant transformation the cell is able to proliferate and survive. This is due to altered cell metabolism and the presence of altered genetic changes that maintain the cell survival. Metabolism was considered an innocent bystander that was a consequence of the increased nutrient requirement for the survival and proliferation of haematological malignancies. The interdependency of metabolism and cellular mechanisms such as autophagy are becoming more evident and important. This interdependence contributes to increased cancer progression and drug resistance. In this paper we aim to discuss autophagy, how it pertains to metabolism in the context of hematologic malignancies, and the implications for therapy.

## 1. Introduction

Autophagy was first described in the 1960s but its importance in various physiological conditions in addition to the basic molecular understanding of autophagy has only come into focus in the last decade. The word autophagy is derived from Greek: *auto*, meaning “self” and *phagy*, “to eat.” This term was coined due to the process by which cellular components are degraded through the lysosomal enzymatic pathway providing a cell with essential amino acids, nucleotides, and fatty acids that enable production of the elements required for energy and macromolecule production [1, 2]. Normal cells engage in autophagy as a means to survive disruptions in nutrient and growth factor availability. It also serves to eliminate damaged organelles and proteins to prevent accumulation. This prevents them from becoming toxic to the cell. If autophagy is prolonged to a point where normal cell function is compromised, cells undergo cell death either through apoptosis or by autophagy itself. One of the main inducers of autophagy is metabolic stress, and understanding the relationship between autophagy and metabolism could lead to better therapeutic strategies in treatment of haematological malignancies. 

## 2. Regulation of Autophagy

Autophagy is characterized by cytoplasmic constituents sequestered into double-membraned vacuoles called autophagosomes. Autophagosomes then fuse with lysosomes (autolysosomes). Autolysosomes degrade cellular components releasing required nutrients to the cell. The regulation of autophagosome and autolysosome structures requires both positive and negative signaling pathways. The discovery in yeast of autophagy-related genes (ATGs) has provided greater understanding of these signaling pathways involved in autophagosome formation [3, 4]. The initial signal to form autophagosomes is by the class III phosphatidylinositol (PI) 3 kinase complex consisting of Beclin1/Atg6, p150hVSp35, and class III PI3K (Vps34). This complex is required for formation of the preautophagosome structures [5]. Binding of ATG14, UVRAG (protein product of ultraviolet radiation resistant gene), and AMBRA1 (activating molecule of Beclin1-regulated autophagy) to the PI3K-III complex further increases the formation of autophagosomes allowing cells to regulate the amount of autophagy. AMBRA1 has also been shown to be a target of ULK1 [6]. ULK, TOR, FIP, Atg13, and AMPK represent molecules in the autophagy signaling network. The formation of the Beclin1 complex is important to autophagosome formation. This process is negatively regulated by binding of Bcl-2 family members such as Bcl-x_L_ to Beclin1 preventing Beclin1 binding to the PI3K-III complex and thereby reducing autophagy [5, 7]. 

Following PI3K-III complex induction of preautophagosome structures, a series of ATG proteins build autophagosomes using an ubiquitin-like mechanism. There are two ubiquitin-like mechanism used in autophagosome formation [8]. The first reaction is the ubiquitin-like protein ATG12 forming a conjugation to ATG5 via the E1 like protein Atg7 and E2-like protein ATG10 or ATG3. ATG16 then binds to the complex and integrates into the autophagosome membrane. The second reaction is the formation of the autophagosome membrane by ubiquitin-like protein LC3 (ATG8) conjugation with phosphatidyl ethanolamine (PE). This is regulated by ATG4 cysteine protease cleavage of LC3 at the C-terminus that facilitates lipidation of LC3 and generating LC3-PE conjugates. When both LC3-PE conjugates and Atg5-Atg12-Atg16 protein complex are localized to the autophagosome, the formation of autophagosomes is complete [8–10]. This process is regulated by acetylation of ATG-3 by histone acetyltransferase Esa-1 suggesting that protein acetylation regulates autophagy [11]. 

Autophagosome formation is negatively regulated by the mammalian target of rapamycin (mTOR) pathway, a nutrient-sensing kinase pathway. Under growth conditions, the mTOR pathway regulates cell growth and survival but under nutrient starvation conditions, the mTOR pathway is inhibited allowing for induction of autophagy [5]. There are two different mTOR complexes: mTORC1 and mTORC2 [12]. mTORC1 complex contains mTOR and regulatory associated protein of mTOR (Raptor). The mTORC2 complex contains mTOR and rapamycin insensitive companion of mTOR (Rictor) [12, 13]. In nutrient rich conditions, the PI3K/AKT signaling pathway activates mTOR allowing formation of mTOR complexes and suppresses autophagy. This is through the mTORC1 where it binds to ULK1/2 (orthologue of yeast ATG1), mATG13, FIP200, and Atg101. Upon formation of this complex, mTOR phosphorylates ULK1 and ATG13 preventing ULK1 activation thereby blocking autophagosomes formation. In nutrient limiting conditions, the LBK/AMPK pathway is activated blocking mTOR activation. This is achieved by preventing mTORC1 binding to ULK complexes. This leads to ULK1 phosphorylation of ATG13 and FIP200 and autophagosome formation [12–14]. Alternatively, growth factor deprivation leads to activation of glycogen synthesis kinase-3 (GSK-3) that phosphorylates an acetyltransferase TIP60 which in-turn acetylates and activates ULK1 [15]. This leads to autophagy. Besides mTOR signaling, rubicon is also a negative regulator of autophagy and the normal maturation of the autophagosome. It thus serves as a brake in the autophagy process [16].

Autolysosomes are formed when autophagosomes and the lysosomes fuse. This allows the degradation of autophagosomal cargo. The lysosome proteins LAMP1 and LAMP2 are found in autolysosomes and are involved in degradation. In addition, the presence of cargo receptors or chaperone proteins, such as p62/SQSTM1 and NBR1, are responsible for sequestration of the ubiquitinated proteins into autophagosomes and autolysosomes [17]. Interactions between these autophagic adapters and the autophagosomal marker protein LC3, are required for efficient selective autophagy. The best characterized is p62/SQSTM1 that is responsible for delivering ubiquitinated proteins to autolysosomes for degradation [18]. Inefficient autophagic degradation of p62 leads to accumulation of ubiquitinated aggregates. This process is inhibited by reactivation of the mTOR pathway that causes the conversion of autolysosomes back to lysosomes. Thus, autophagy is a tightly regulated process that breaks down cellular constituents in response to cellular stress.

## 3. Autophagy and Normal Haematopoiesis

In the normal development of the erythrocyte, the reticulocyte is enucleated but retains its organelles. Through the process of autophagy, it then loses its organelles to become a circulating red blood cell [19]. Nix, also known as BNIP3L, has been demonstrated to play a role in this process through regulation of mitochondrial clearance [20]. Chemical differentiation of the K562 CML cell line also demonstrates a role for autophagy in megakaryocyte differentiation [21]. In lymphocytes autophagy proteins Atg5, Beclin1, and LC3 are upregulated in early thymocyte development and T-cell activation but downregulated in the mature CD4+ and CD8+ T cells [22]. Loss of autophagy gene Atg5 is important for B-cell survival during development. Loss of this gene leads to inefficient B cell development characterized by increased cell death [23]. Autophagy is also known to regulate haematopoietic stem cells (HSCs) that are critical for normal hematopoiesis [24–26]. Recent studies showed that autophagic gene ATG7 is an essential regulator of adult HSCs maintenance since haematopoietic stem and progenitor cell lacking ATG7 expression have increased proliferation and DNA damage [27, 28]. This confirms that autophagy is an important regulator of early development, homeostasis, and maintenance of haematopoiesis.

## 4. Autophagy in Haematologic Malignancies

The role of autophagy in haematologic malignancies is controversial [29, 30]. Autophagy has been shown to be either tumor promoting or tumor suppressive. Studies demonstrating a tumorigenic role of autophagy suggesting cancer cells can adapt and thrive to harsh environmental conditions such as low nutrients, growth factor deprivation, and metabolic stress because of autophagy [31–33]. This is due to the ability of autophagy to protect against apoptotic signaling through the degradation of damaged mitochondria, aggregated proteins, and pathogens within a cell [30, 34, 35]. However, this distinct role of autophagy during tumor progression is cancer type specific depending on the developmental context and stage of cancer [30, 34, 35]. In normal haematopoeisis, autophagy regulates homeostasis. However, when this balance is disturbed the initiation of myelodysplastic syndrome (MDS) and acute myeloid leukemia (AML) ensues [36, 37]. In addition, autophagy plays a role in cell survival in haematologic malignancies. This is illustrated by development of resistance to therapy such as chronic myelogenous leukemia resistance to imatinib [38].

**Figure 1:**
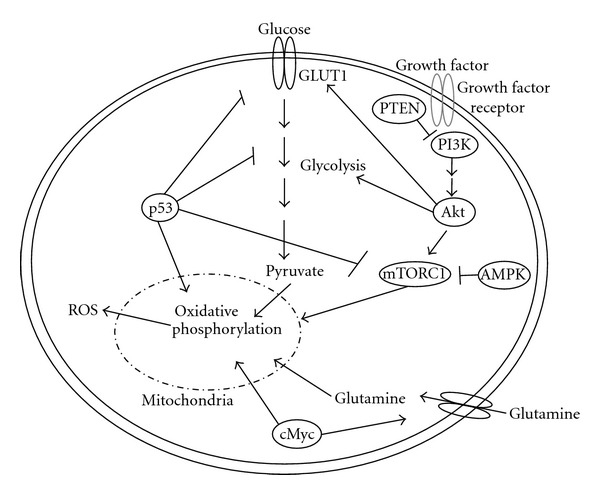
Metabolic signaling regulation. Glucose uptake in cells is regulated by Glut1 transporters that increase glycolysis and oxidative phosphorylation. This is enhanced by cMyc regulation of glutamine uptake in cells. Growth factors influence metabolism through activation of the PI3K/AKT/mTOR signalign pathway contribute to increased glycolysis and oxidative phosphorylation. This is inhibited through PTEN. Under glucose limiting conditions, AMPK is activated inhibiting the mTOR signaling pathway. In addition, p53 activaiton inhibits glycolysis, and the mTOR pathway but increases oxidative phosphorylation. ROS increases through inefficient oxidative phosphorylation at the mitochondria.

Besides the role of autophagy in tumour progression, there is also evidence that supports a tumor suppressive role of autophagy. Beclin1, an autophagy gene, is found to be a haploinsufficient tumour suppressor in mice and is mono-allelically deleted in human breast, ovarian, and other tumors but not in haematologic malignancies [39, 40]. p53 and PTEN are the most commonly mutated tumor suppressor genes and regulate autophagy in haematologic malignancies [41]. Through increased expression of autophagy genes such as DRAM, p53 increases autophagy where mutant p53 fails to increased autophagy gene expression and contributes to cell survival. Indeed, altered expression of autophagic genes Beclin1 or LC3 are considered prognostic markers in many tumours including non-Hodgkin lymphoma [42–44]. PTEN inhibits the PI3K/AKT pathway leading to decreased activation of the mTOR signaling pathway (Figure 1) and increased autophagy [45]. In contrast, mutant PTEN suppresses autophagy levels [46]. Autophagy can also function to promote apoptosis or induce cell death. Autophagic cell death has been demonstrated under various conditions in hematologic malignancies but the mechanisms that govern autophagy leading to tumour suppressive roles being unknown.

## 5. Autophagy and Metabolism 

Autophagy is negatively regulated by growth factors, amino acids, and glucose signals leading to the nutrient responsive mTOR-signaling pathway [47] (Figure 1). Autophagy is regulated by adenosine monophosphate kinase (AMPK) via the mammalian target of rapamycin (mTORC1) pathway. AMPK senses changes in lipids and glucose to function as a metabolic sensor. It restores energy balance in the adenosine monophosphate (AMP) and adenosine triphosphate (ATP) ratio by the LKB1-AMPK activation. In AML the LKB1/AMPK pathway plays a tumor suppressor role through repression of mTOR-dependent mRNA translation [37]. Similarly, tumour necrosis inducing apoptosis ligand (TRAIL) is involved in apoptosis via intrinsic and extrinsic pathways. However, certain blood cancers such as chronic lymphocytic leukemia (CLL) are resistant to TRAIL-induced apoptosis. This could be due in part to TRAIL-induced cytoprotective autophagy. Thus, targeting autophagy genes such as Beclin1 and Atg-5 enables TRAIL induced apoptosis [48, 49]. Autophagy may play a role in the progression of low-risk MDS to AML by protecting the cells from extensive reactive oxygen species (ROS) induce damage from altered metabolism [37].

ROS play an important role in regulating metabolism and autophagy. ROS consist of unpaired electrons molecules such as superoxide (O_2_^−^), hydrogen peroxide (H_2_O_2_), hydroxyl radical (OH^−^), nitric oxide (^•^NO), peroxynitrite (ONOO^−^), and nitrogen dioxide radical (^•^NO_2_) [50–52]. Although ROS is formed from normal metabolism mainly from the mitochondria (Figure 1) and plays an important role in cell signaling and homeostasis leading to cell survival, ROS levels can increase causing irreversible oxidative damage leading to impaired metabolism and cell death [51, 53–55]. It has been documented that many chemotherapeutic agents raise levels of intracellular ROS [54, 55]. The essential *role of mitochondria* in generation of ROS and regulating tumorigenesis is implicated in many cancers including hematologic malignancies [36, 56]. Metabolic and oxidative stress also increases autophagy and blockage of ROS production or use of free radical scavengers inhibits autophagy. The mechanism of ROS induced autophagy is unclear but several possible mechanisms have been proposed. The cysteine protease Atg4 could be oxidized on a cysteine residue located near the active site, critical for its regulation. Atg4 regulates the reversible conjugation of Atg8 (LC3 in mammals) to the autophagosomal membrane, required for autophagosome formation [57]. Starvation-induced oxidative inactivation of ATG4 promotes lipidation of ATG8, facilitating autophagosome formation [57]. ROS accumulation could also be caused by selective autophagic degradation of catalase. Catalase degradation subsequently caused further ROS accumulation [58]. Other potential mechanisms for ROS regulation of autophagy could be through activation of transcription factor activity, leading to altered gene expression [59]. Indeed, autophagy genes are up-regulated in response to oxidative stress in yeast, and ROS induce Beclin1 and ATG-7 expression in different cancer cells. We have demonstrated that mitochondria are an important source of ROS leading to autophagy since oxidative phosphorylation inhibitors could induced autophagy mediated by ROS [51]. ROS induced autophagy has been shown to lead to cytoprotection and autophagic cell death. It was demonstrated that histone deacetylase inhibitor, SAHA induced autophagy and increased ROS leading to a cellular prosurvival mechanism in Jurkat T-cells [60]. In addition, FTY720, an immunosuppressive drug, induced cytoprotective autophagy in ALL [61]. In contrast, many chemotherapeutic drugs induce oxidative stress causing autophagic cell death. For example, increases in ROS, autophagosome formation and cell death have been detected upon Brevinin-2R treatment in Jurkat and BJAB (B-cell lymphoma) cells [62]. Another study showed that natural compound eupalinin-induced autophagic cell death through increased ROS in human leukemia cells [63, 64]. Taken together, increased cellular ROS production by therapeutic drug initiates a stress response leading to either cell survival or cell death. 

Both oncogene activation and tumor suppressor gene loss can all lead to deregulation of metabolic pathways such as glycolysis, pentose phosphate pathway, and lipid and energy metabolism. Cancer growth is dependent on functional mitochondria that are using glutamine as their major source of fuel for the citric acid cycle and the generation of NADPH and lipid synthesis [65]. The oncoprotein MYC activation is common in haematologic malignancies such as Burkitts lymphoma and AML. MYC upregulates glutamine transporters (Figure 1) and glutaminolysis, which increase ammonia production and autophagy protecting the cells from apoptosis [66, 67]. NF*κ*B activation is common in a variety of B-cell neoplasm including diffuse large B-cell lymphoma. Sommermann et al. showed that inhibition of NF*κ*B-induced cell death via the PI3K pathway and GLUT1 by restricting glucose transport [68]. To this end, it has been demonstrated that autophagy inhibitors in combination with NF*κ*B induce a “metabolic crisis” and cell death [68]. Activating mutations in the oncogene Ras induce autophagy possibly through a novel AKT1-GLI3-VMP1 pathway [69]. This is essential for overcoming metabolic stress by impaired acetyl-CoA production leading to survival and tumor growth [70]. Overall, this demonstrates the interdependence of oncogene-mediated metabolic pathways and autophagy in response to cellular stress and cancer progression. 

Besides oncogenes, tumour suppressors also regulate autophagy. Otto Warburg first observed that cancer cells undergo aerobic glycolysis due to lack of mitochondrial oxidative phosphorylation. The tumor suppressor, p53, positively regulates oxidative phosphorylation via synthesis of cytochrome c oxidase (COX-2) and downregulates glycolysis via transcription of TP53-induced glycolysis and apoptosis regulator (TIGAR) [71] (Figure 1). Loss of p53 enhances aerobic glycolysis resulting in more aggressive cancer phenotypes. p53 is often loss in cancer thus maybe an important genetic change contributing to the “Warburg effect.” p53 is known as a regulator of apoptosis, however its role in coordination of nutrient utilization in order to preserve cell survival is equally important. TIGAR is a direct transcriptional target of p53 and alters cellular use of glucose. TIGAR shares sequence homology with the bisphosphatase domain of 6-phosphofructo-2-kinase/fructose-2-,6-bisphosphataseand dephosphorylate fructose 2,6-bisphosphate reducing the levels of this metabolite. In addition, TIGAR suppresses ROS levels and autophagy. In the glycolytic pathway 6-Phosphofructo-1-kinase (PFK-1) converts fructose 6 phosphate to fructose1, 6-bisphosphate. This in turn activates PFK-1 mediated by TIGAR and leads to inhibition of glycolysis. p53 also modulates another enzyme later in the glycolytic pathway, phosphoglyceratemutase (PGM). Wild type p53 downregulates PGM whereas p53 mutation increases its activity and leads glycolytic flux. In addition to regulation of glycolytic enzymes via TIGAR and PGM, p53 is important in the regulation of glucose transport. p53 can also down regulate glucose transporter expression leading to a reduction in intracellular glucose. Glucose transporter 1 (GLUT1) and glucose transporter 4 (GLUT4) are directly repressed at the gene promoter by p53 (Figure 1). This is important in autophagy since autophagy is activated by metabolic stress (glucose deficit) leading to degradation and recycling of cellular substrates that support metabolism and promote survival and tumor growth. p53 acts as a key regulator element autophagy through regulation of glycolytic pathway and hence metabolic stress.

**Figure 2:**
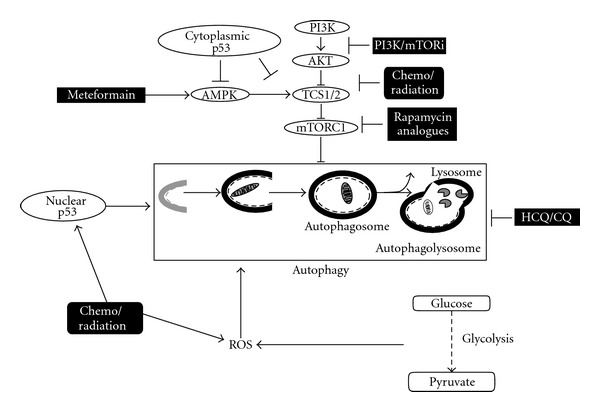
Signaling pathways regulating autophagy and their inhibitors. Autophagy is a catabolic process that results in the autophagosomic-lysosomal degradation of bulk cytoplasmic contents. The kinase mTOR is a critical regulator of autophagy induction, with activated mTOR (PI3K/Akt) suppressing autophagy. AMPK-signaling negatively regulates mTOR signaling therefore promoting autophagy. ROS stress is an important inducer and regulator of autophagy generated by reduced oxidative phosphorylation and increased glycolysis. Nuclear 53 induces autophagy through transcriptional regulation of multiple genes whereas cytoplasmic p53 inhibits autophagy by blocking the mTOR pathway. Autophagy inhibitors chloroquine (HCQ/CQ) and metformin are under clinical investigation. Many chemotherapy/radiation therapies induce autophagy through ROS, inhibition of the mTOR pathway or nuclear p53 whereas PI3K/mTor inhibitors or rapamycin analogues specifically block the mTOR signaling pathway leading to autophagy.

The role of p53 in regulating autophagy through metabolism is complicated. It regulates through cellular location and by transcriptional dependent and independent mechanisms. Nuclear localization of p53 enables activation of AMPK which then leads to autophagy. A fine balance between nuclear and cytoplasmic p53 is responsible for autophagy homeostasis [72]. Nuclear p53 induces autophagy (Figure 2) through upregulation of mTOR pathway regulators. Under metabolic stress, basal p53 expression regulates multiple detoxifying pathways such as upregulation of antioxidant targets such as GPX1, MnSOD, ALDH4, and TPP53INP1 [73–77]. In addition, p53 target genes, *sestrin1 *and *sestrin2*, have been identified as a connection between p53 activation and mTORC1 activity [73, 78]. p53 exerts the antioxidant effect via inducing Sestrin expression in response to DNA damage and oxidative stress which leads to inhibition of mTORC1 activity and autophagy. Sestrins inhibit mTORC1 activity by interacting with mTOR pathway suppressors AMPK, TSC1, and TSC2 [78]. In contrast, cytoplasmic p53 inhibits autophagy mediated by activation of mTOR downstream signaling [79] (Figure 2). In addition, the mTOR pathway activates MDM2, the major ubiquitin ligase that reduces nuclear p53 expression [80]. Cytoplasmic p53 also binds to high mobility group box 1 (HMGB1) preventing formation the HMGB1/Beclin 1 complex, and inhibiting autophagy [81]. Beclin1 also controls the protein stabilities of ubiquitin-specific peptidases, USP10 and USP13, by regulating their deubiquitinating activities. Since USP10 mediates the deubiquitination of p53, regulating deubiquitination activity of USP10 and USP13 by Beclin1 provides a mechanism for Beclin1 to control the levels of p53 [82]. Moreover, p53 inhibition was found to promote cell survival in response to glucose starvation through autophagy [83]. All these results suggest that the autophagy induced by p53 deletion in tumors provide a survival advantage to malignant cells in response to unfavorable conditions. Taken together, p53 signaling regulates autophagy in response to metabolic stresses. 

All these oncogenes and tumor suppressors play important roles in development and progression of hematological malignancies. Metabolic alterations are also a common feature in hematological malignancies. Thus, it is reasonable to suggest that these alterations regulated autophagy in hematological malignancy contributing to tumor survival and suppression. There are, however, many unanswered questions. What autophagy supplied substrates are essential for sustain metabolism? What affect do changes in metabolism and upstream signaling pathways have on autophagy in normal hematological stem cells or other immune cells? Nevertheless, targeting of autophagy regulatory pathways could provide treatments for hematological malignancies through either blocking or inducing autophagy.

## 6. Targeting Autophagy and Metabolic Deregulation in Hematological Malignancies

Chemotherapy or radiotherapy can both induce autophagy as a protective mechanism and lead to therapy resistance directly via mTOR inhibition and others indirectly by cytotoxic stress [84]. It may also cause chemoresistance by interfering with ROS activation that is the mechanism by which many chemotherapeutic agents function [84] (Figure 2). Inhibition of the proteasome induces autophagy and may pose reason for concern and resistance to therapy [85]. Hydroxychloroquine and chloroquine are known inhibitors of autophagy. They are also known antimalarials and thus clinically relevant compounds [86] (Figure 2). These agents have shown efficacy in targeting p53 loss induced autophagy and Myc induced autophagy in pre-clinical models [87, 88]. Thus, the rational combination of an autophagy inhibitor chloroquine is being tested in clinical trial with bortezomib, a proteasome inhibitor, and cyclophosphamide in relapsed refractory multiple myeloma in a nonrandomized open label phase II clinical trial to determine if the combinatorial effects have clinical efficacy (http://clinicaltrials.gov/ct2/show/NCT01438177). 

Many anticancer agents induce cell death through autophagy in hematologic malignancies instead of through cell survival by altering metabolism (Figure 2). For instance, arsenic trioxide (As_2_O_3_) a potent antimetabolite exhibited potent antitumor effects through autophagic cell death in leukemic cell lines and primary leukemic progenitors from acute myelogenous leukemia (AML) patients [89–91]. Moreover, arsenic trioxide-induced autophagy through inhibiting the mTOR pathway contributes to degradation of the PML/RARA fusion protein in acute promyelocytic leukemia (APL) [91, 92]. In addition, mTOR inhibitor NVP-BEZ235 treatment in T-ALL cells caused suppressing PI3K/Akt/mTOR signaling and induced autophagic cell death [93]. mTOR inhibitor RAD001 (Everolimus), also induced cell death by inducing autophagy in an *in vivo* model of childhood ALL [94, 95]. Resveratrol (RSV) is another attractive agent that induces autophagic cell death by inhibiting the AMPK/mTOR pathway in CML cells [96, 97] Histone deacetylase inhibitors are another class of agents that can be used to target autophagy. Although currently approved for the use of cutaneous T-cell lymphomas, suberoylanilide hydroxamic acid (SAHA) has been found to have activity in imatinib refractory CML. In addition, there is evidence to suggest that chloroquine maybe synergistic with SAHA in this clinical scenario [98, 99]. Sphingolipids can also induce autophagy leading to increased apoptosis in leukemias and changes in sphingolipid metabolism have been observed in hematological malignancies [100, 101]. Thus, targeting metabolic signaling pathways leading to autophagy could be an effective treatment of malignant hematologic disorders.

Finally, metformin, a biguanide, used to treat diabetes has been suggested as a potential anticancer drug. Metformin is a known LKB-1/AMPK activator (Figure 2). In melanoma, metformin was found to induce autophagy by increased expression of Beclin1, and accumulation of LC-3 secondary to mTOR inhibition leading to cell death [102]. Similar effects have recently been described in lymphoma [103]. Metformin-induced activation of AMPK and inhibition of mTOR is AKT independent manner [103]. This lead to attenuated cell growth via induction of autophagy. The effect was evident in combination with doxorubicin versus single agent therapy and was reversed by autophagic inhibitor 3-methyladenine [103]. In T-ALL, metformin was found to have a significant antileukemic effect [104]. Metformin induced autophagy as evidenced by electron microscopy and increase in the LC3-II protein possibly contributing to cell death. 

The major issue remains in hematological malignancies therapy as to whether induced or inhibited autophagy. The context of metabolism in cancer cells might be the key to this question and will govern the development of innovative metabolic therapies for hematological malignancies in the future. 

## 7. Conclusion

The role of autophagy in cancer is multifaceted and its implication in metabolism is no different. This being said we are making headway in its understanding; however, there is more research required to understand the interactions between these currently distinct entities that are now merging in the pathogenesis of cancer. In hematologic malignancies it plays a role in pathogenesis, homeostasis, survival, and even cell death. An emerging role for metabolism has shed light on the interconnection between metabolism and autophagy. Metabolisms effect on autophagy is still ambiguous; it may lead to cell survival or cell death. Clinical evidence does support a role for metformin as an anticancer agent. It is also being looked at in the context of cancer prevention. In leukemia, it may be a realistic thought to use emerging technologies for metabolic profiling and treat patients in a personalized manner. The question that remains unanswered is whether to inhibit or activate autophagy as a treatment of hematological malignancies.
